# Exploiting Broad-Spectrum Chimeric Lysin to Cooperate with Mupirocin against Staphylococcus aureus-Induced Skin Infections and Delay the Development of Mupirocin Resistance

**DOI:** 10.1128/spectrum.05050-22

**Published:** 2023-05-01

**Authors:** Xiao-chao Duan, Xin-xin Li, Xiang-min Li, Shuang Wang, Fen-qiang Zhang, Ping Qian

**Affiliations:** a State Key Laboratory of Agricultural Microbiology, Huazhong Agricultural University, Wuhan, China; b College of Veterinary Medicine, Huazhong Agricultural University, Wuhan, China; c Key Laboratory of Preventive Veterinary Medicine in Hubei Province, The Cooperative Innovation Center for Sustainable Pig Production, Wuhan, China; University of Guelph College of Biological Science

**Keywords:** chimeric phage lysin, mupirocin, *S. aureus* infections, antibiotic resistance

## Abstract

Staphylococcus aureus often leads to severe skin infections. However, S. aureus is facing a crisis of antibiotic resistance. The combination of phage and antibiotics is effective for drug-resistant S. aureus infections. Therefore, it is worth exploiting novel antibacterial agents to cooperate with antibiotics against S. aureus infections. Herein, a novel chimeric lysin ClyQ was constructed, which was composed of a cysteine- and histidine-dependent amidohydrolase/peptidase (CHAP) catalytic domain from S. aureus phage lysin LysGH15 and cell wall-binding domain (CBD) from Enterococcus faecalis phage lysin PlyV12. ClyQ had an exceptionally broad host range targeting streptococci, staphylococci, E. faecalis, and *E. rhusiopathiae*. ClyQ combined with mupirocin (2.64 log reduction) was more effective at treating S. aureus skin infections than ClyQ (0.46 log reduction) and mupirocin (2.23 log reduction) alone. Of equal importance, none of S. aureus ATCC 29213 or S3 exposed to ClyQ developed resistance, and the combination of ClyQ and mupirocin delayed the development of mupirocin resistance. Collectively, chimeric lysin ClyQ enriches the reservoirs for treating S. aureus infections. Our findings may provide a way to alleviate the current antibiotic resistance crisis.

**IMPORTANCE** Staphylococcus aureus, as an Enterococcus faecium, Staphylococcus aureus, Klebsiella pneumoniae, Acinetobacter baumannii, Pseudomonas aeruginosa, and Enterobacter species (ESKAPE) pathogen, can escape the elimination of existing antibiotics. At present, phages and phage lysins against S. aureus infections are considered alternative antibacterial agents. However, the development of broad-spectrum chimeric phage lysins to cooperate with antibiotics against S. aureus infections remains at its initial stage. In this study, we found that the broad-host-range chimeric lysin ClyQ can synergize with mupirocin to treat S. aureus skin infections. Furthermore, the development of S. aureus resistance to mupirocin is delayed by the combination of ClyQ and mupirocin *in vitro*. Our results bring research attention toward the development of chimeric lysin that cooperates with antibiotics to overcome bacterial infections.

## INTRODUCTION

Staphylococcus aureus, as the most common commensal bacteria on the skin (1), plays a major part in atopic dermatitis (AD), and more than 90% of AD cases are caused by S. aureus (2, 3). Most studies on S. aureus have been emphasized with the attention being given to the antimicrobial resistance of S. aureus (4). Furthermore, biofilms protect S. aureus from the clearance of antibiotics and the immune system (5).

Currently, phage lysins, such as Staphylococcus phage endolysin SAL-200 (6), antistaphylococcal lysin LSVT-1701 (7), and Streptococcus suis phage lysin CF-301 (8), have been successfully applied to treating clinical bacterial infections. As expected, this has boosted the study of phage lysins and chimeric lysins as new antimicrobial agents in S. aureus-caused infections (9, 10). Notably, previous studies demonstrated that CF-301 has synergistic antibacterial activity against S. aureus with antibiotics *in vitro* and *in vivo* (11). The combination of endolysin MR-10 and minocycline could treat methicillin-resistant S. aureus (MRSA)-induced wound infection (10). Moreover, both phage lysin PlySs2 and chimeric lysin ClyS did not cause the development of S. aureus and S. pneumoniae resistance within 8 days (12, 13). Additionally, the combination of CF-301 and daptomycin delayed the development of S. aureus MW2 resistance to daptomycin after 26 days of passage (14). Although numerous studies have demonstrated that chimeric lysins possess perfect activity against S. aureus *in vivo* and *in vitro* (15–17), there are no reports on a chimeric lysin that yields a surprising broad-spectrum activity and exhibits a synergistic antibacterial effect with antibiotics.

In this study, we constructed a novel chimeric lysin ClyQ, which possesses antibacterial activity against staphylococci, streptococci, Enterococcus faecalis, and Erysipelothrix rhusiopathiae *in vitro*. Our results revealed that ClyQ could significantly remove the biofilms of S. aureus. Meanwhile, ClyQ contributed to treating S. aureus systemic and skin infections. Remarkably, the combination of ClyQ and mupirocin was superior to ClyQ and mupirocin treatment alone for removing S. aureus from the skin and delaying the development of resistance to mupirocin.

## RESULTS

### Construction, expression, and purification of ClyQ.

To solve the limited host range of phage lysins, we constructed a novel chimeric lysin ClyQ. ClyQ was composed of a cysteine- and histidine-dependent amidohydrolase/peptidase (CHAP) domain from S. aureus phage lysin LysGH15 and a cell wall-binding domain (CBD) from E. faecalis phage lysin PlyV12, and the two domains were ligated by a glycine-serine (GGSSGS) linker (Fig. S1A in the supplemental material). The molecular weight of purified ClyQ was approximately 38.0 kDa (Fig. S1B).

### Stability of ClyQ.

Our results showed that the lytic activity of ClyQ could not be significantly changed at 37 to 42.5°C. When ClyQ was incubated at 45°C, the activity of ClyQ began to decrease and was completely lost at 60 min (Fig. S2A), and the protein melting temperature (*T_m_*) of ClyQ was 43.9°C (Fig. S2B). Concentrations of 10, 100, and 1,000 μM Ca^2+^, Mn^2+^, Mg^2+^, and Ba^2+^ could promote the lytic activity of ClyQ. Simultaneously, the strength of 10 μM Zn^2+^ could not affect ClyQ, although 100 and 1,000 μM Zn^2+^ inhibited the lytic activity of ClyQ (Fig. S2C). Furthermore, ClyQ could maintain complete activity at pH 7 to 10. The activity of ClyQ was reduced by approximately 23.3% after incubation in phosphate-buffered saline (PBS; pH 11) for 60 min, and ClyQ had little activity at pH 5 to 6 (Fig. S2D).

### ClyQ exhibits excellent antibacterial effects *in vitro*.

**FIG 1:**
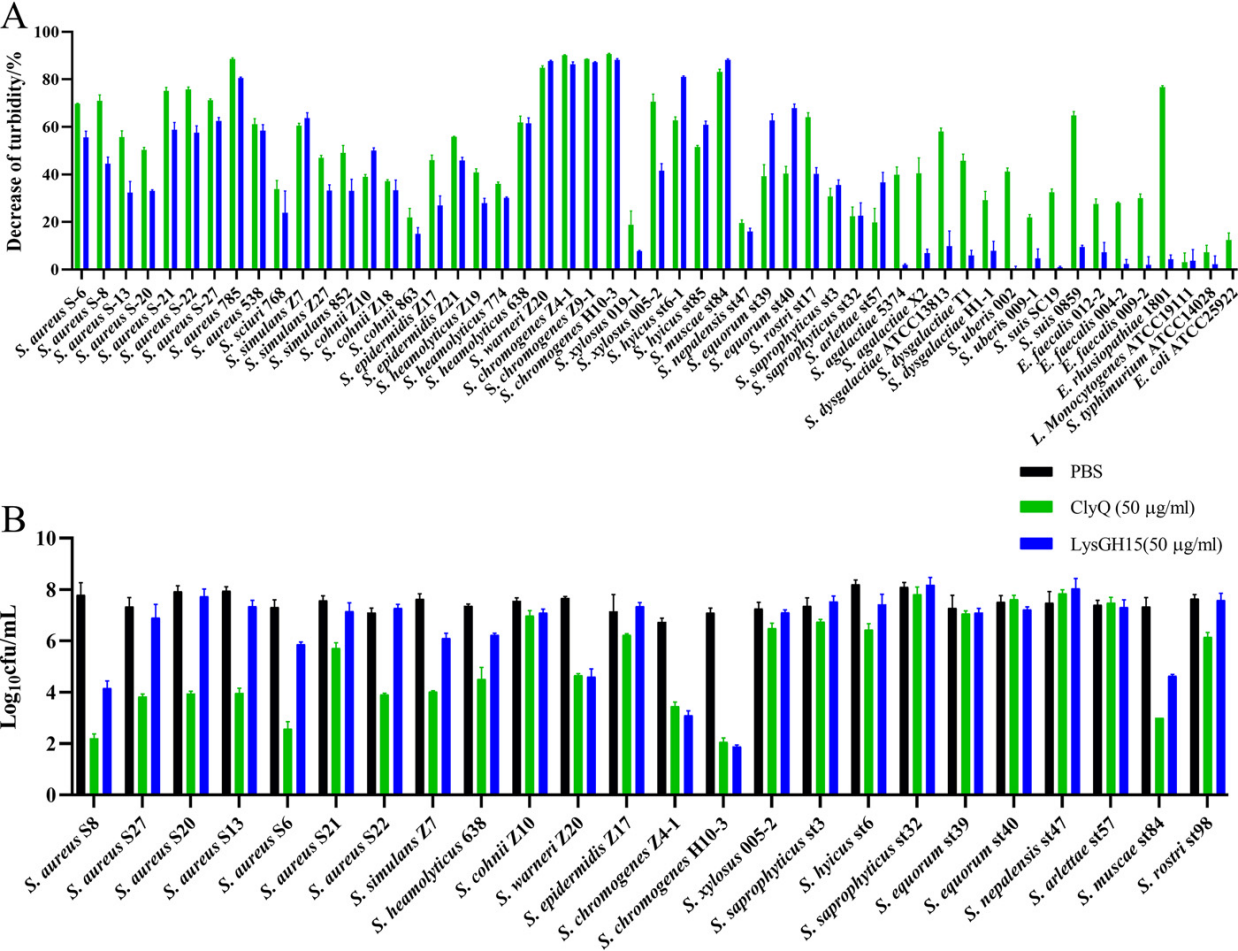
The host range and antistaphylococcal activity of chimeric lysin ClyQ and S. aureus phage lysin LysGH15 *in vitro*. (A) Host range of ClyQ and LysGH15. Different bacterial species (including staphylococci, streptococci, E. faecalis, *E. rhusiopathiae*, L. monocytogenes, E. coli, and *S.* Typhimurium) were used to determine the host range of ClyQ and LysGH15. (B) The antistaphylococcal activity of ClyQ and LysGH15. Different species of Staphylococcus (including S. aureus, *S. simulans*, *S. cohnii*, S. epidermidis, *S. heamolyticus*, *S. warneri*, *S. chromogenes*, *S. xylosus*, *S. muscae*, *S. nepalensis*, *S. equorum*, *S. rostri*, *S. saprophyticus*, *S. arlettae*, and *S. hyicus*) were used to test the antistaphylococcal activity of ClyQ and LysGH15. All experiments were repeated three times. The error bars show the standard deviations (SD).

LysGH15, as a S. aureus phage lysin, possesses remarkable lytic activity against S. aureus (18). ClyQ shares the same CHAP domain as LysGH15. Thus, the host range and antibacterial activity of ClyQ were compared with those of LysGH15. As shown in Fig. 1A, 9 strains of S. aureus were sensitive to ClyQ and LysGH15, and the turbidity reduction ratio of ClyQ ranged between 50.29 and 88.51% for these S. aureus strains. For other staphylococci (including *S. simulans*, *S. cohnii*, S. epidermidis, *S. heamolyticus*, *S. warneri*, *S. chromogenes*, *S. xylosus*, *S. muscae*, *S. nepalensis*, *S. equorum*, *S. rostri*, *S. saprophyticus*, *S. arlettae*, and *S. hyicus*), the turbidity reduction ratio of ClyQ was in the range of 18.84 to 90.72% and that of LysGH15 was in the range of 14.96 to 88.23%. For streptococci (including S. agalactiae, S. dysgalactiae, *S. uberis*, and S. suis), the turbidity of these strains was reduced by 21.89 to 64.79% (Fig. 1A). For E. faecalis and *E. rhusiopathiae*, the optical density at 600 nm (OD_600_) values could be decreased by ClyQ, but Listeria monocytogenes, Escherichia coli, and Salmonella enterica serovar Typhimurium were not sensitive to ClyQ (Fig. 1A). Additionally, LysGH15 could not lyse the streptococci E. faecalis, *E. rhusiopathiae*, Listeria monocytogenes, Escherichia coli, and Salmonella Typhimurium strains.

At the same time, we evaluated the antibacterial activity of ClyQ and LysGH15 against staphylococci *in vitro* because staphylococci are the primary opportunistic pathogens in the skin (19). Specifically, ClyQ and LysGH15 could reduce the visible bacteria number of S. aureus in bacteria suspensions by 1.86 to 5.59 log and 0.00 to 3.63 log, respectively (Fig. 1B). Moreover, other staphylococci strains were reduced by 0.00 to 5.04 log and 0.00 to 5.21 log, respectively (Fig. 1B). Our results demonstrate that ClyQ possesses a broad host range and exceptional lytic activity.

**FIG 2:**
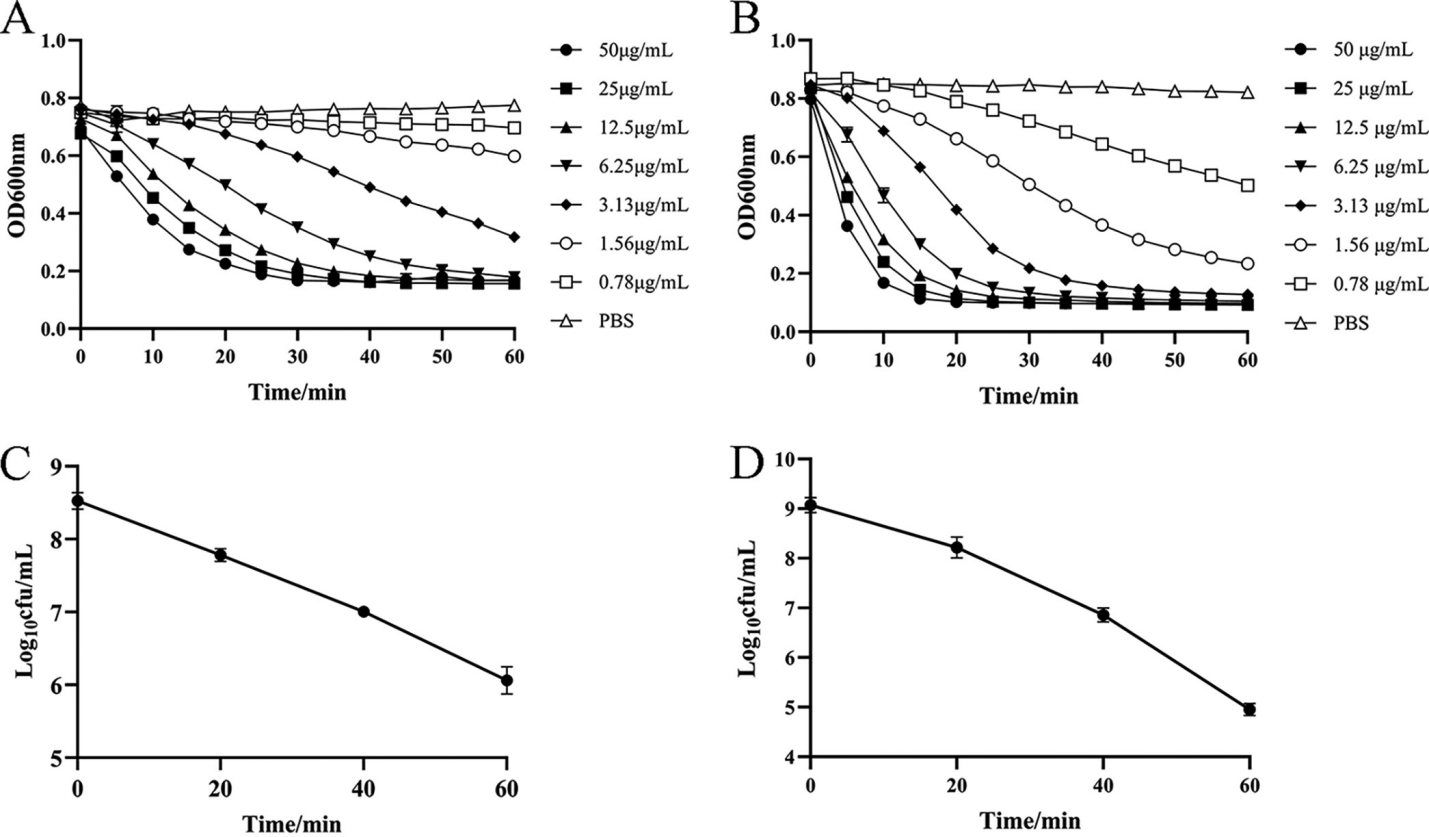
Bactericidal activity of ClyQ against S. aureus ATCC 43300 and S3. (A and B) Lytic activity of ClyQ against S. aureus ATCC 43300 (A) and S3 (B) at different strengths (50, 25, 12.5, 6.25, 3.13, 1.56, 0.78, and 0 μg/mL) within 60 min. (C and D) Time-dependent bactericidal activity of ClyQ (50 μg/mL) against S. aureus ATCC 43300 (C) and S3 (D) within 60 min. All experiments were repeated three times. The error bars show the standard deviations (SD).

We examined the lytic activity of ClyQ under various times and concentrations. Our data indicated that ClyQ could lyse S. aureus ATCC 43300 and S3 in a dose-dependent manner (Fig. 2A and B). At the same time, ClyQ (50 μg/mL) could continuously reduce the visible number of ATCC 43300 and S3 within 60 min (Fig. 2C and D). Specifically, after 60 min of incubation, the bacterial numbers of ATCC 43300 and S3 in the ClyQ-treated bacteria suspensions were reduced by 2.46 and 4.12 log, respectively (Fig. 2C and D).

Furthermore, the MIC of ClyQ against S. aureus strains ranged from 4 to 16 μg/mL and that of *S. equorum*, *S. hyicus*, *S. muscae*, *S. rostri*, S. sciuri, *S. simulans*, *S. cohnii*, *S. simulans*, and *S. warneri* ranged from 2 to 16 μg/mL. Meanwhile, the MIC for the *S. arlettae*, *S. nepalensis*, *S. saprophyticus*, and *S. heamolyticus* strains was greater than 128 μg/mL (Table 1).

**TABLE 1:** MIC values of ClyQ

Strains	Species of strains	MIC (μg/mL)
st57	*S. arlettae*	>128
st84	*S. muscae*	2
st47	*S. nepalensis*	>128
st98	*S. rostri*	1
st74	*S. saprophyticus*	>128
st60	*S. saprophyticus*	>128
Z4	S. sciuri	8
Z7	*S. simulans*	2
Z10	*S. cohnii*	8
Z27	*S. simulans*	8
Z20	*S. warneri*	16
638	*S. heamolyticus*	>128
ATCC 43300	S. aureus	16
ATCC 29213	S. aureus	4
S3	S. aureus	8
st95	S. aureus	4
st18	S. aureus	8
st76	S. aureus	4
st80	S. aureus	4
st39	*S. equorum*	2
st40	*S. equorum*	2
st156	*S. equorum*	>128
st6	*S. hyicus*	2
st85	*S. hyicus*	8
st96	*S. hyicus*	8

### ClyQ effectively clears the biofilms of S. aureus.

**FIG 3:**
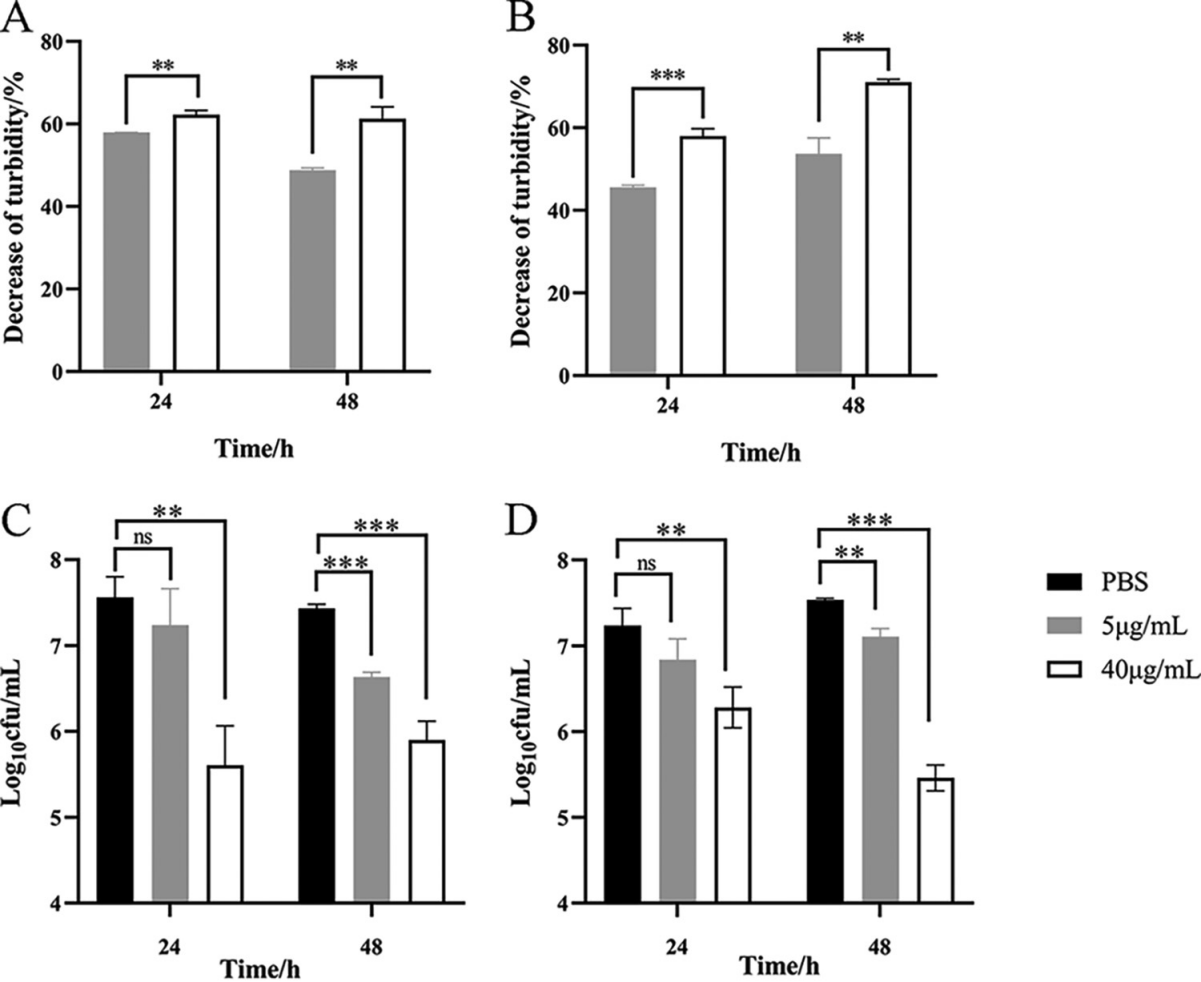
The antibiofilm activity of ClyQ. (A and B) The biofilms of S. aureus ATCC 43300 (A) and S3 (B) were removed by treatment with ClyQ (5 and 40 μg/mL) at 37°C for 1 h. (C and D) Viable cell counts of S. aureus ATCC 43300 (C) and S3 (D) biofilms treated with ClyQ (5 and 40 μg/mL) at 37°C for 1 h. Significant differences between the PBS groups and the 5 μg/mL ClyQ or 40 μg/mL ClyQ group were determined by Student’s *t* test; ns, not significant; **, *P* < 0.01; ***, *P* < 0.001. Error bars show the standard deviations (SD).

**FIG 4:**
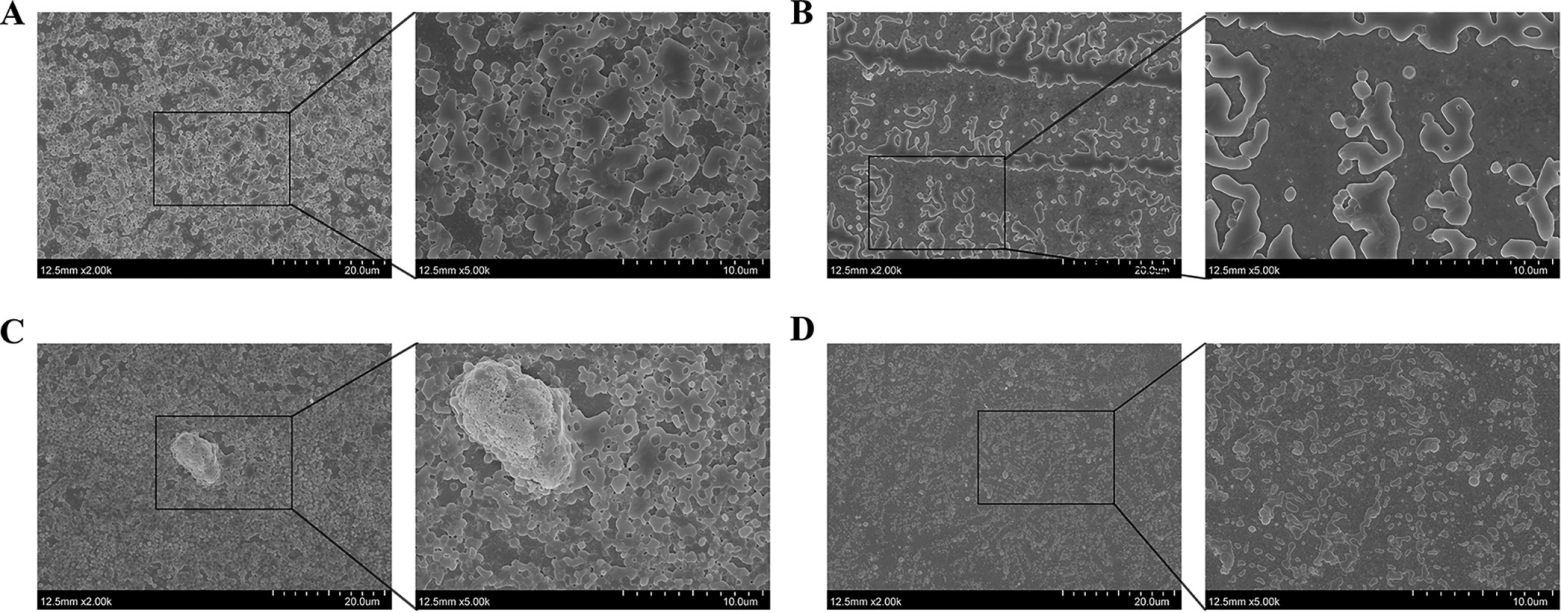
Field emission scanning electron microscope images of biofilms formed by S. aureus ATCC 43300 and S3. The magnifications of the images are ×2,000 (left images) and ×5,000 (insets). The 48-h biofilms formed by S. aureus S3 were treated with PBS (A) and ClyQ (B), and the 48-h biofilms formed by S. aureus ATCC 43300 were treated with PBS (C) and ClyQ (D).

S. aureus can colonize and form biofilms on skin infection sites, contributing to antibiotic resistance (20, 21). As a result, crystal violet (CV) staining and colony count methods were used to evaluate the effect of ClyQ on clearing S. aureus-formed biofilms. On the one hand, the results of CV staining indicated that 24-h and 48-h biofilms of S. aureus ATCC 43300 were cleared after ClyQ (40 μg/mL) treatment by 62.27 and 61.28%, respectively (Fig. 3A), and 40 μg/mL ClyQ could clear 24-h and 48-h biofilms of S. aureus S3 by 58.01 and 71.04%, respectively (Fig. 3B). On the other hand, as shown in Fig. 3C, ClyQ at a concentration of 40 μg/mL could decrease the number of sessile S. aureus ATCC 43300 in biofilms by 1.95 log (24-h biofilm) and 1.54 log (48-h biofilm). The number of sessile S. aureus S3 was decreased after treatment with 40 μg/mL ClyQ by 0.95 log (24-h biofilm) and 2.07 log (48-h biofilm) (Fig. 3D). In addition, field emission scanning electron microscope (FESEM) images showed that ClyQ could remove sessile bacteria and biomass in biofilms (Fig. 4). These data indicate that ClyQ can effectively clear S. aureus-formed biofilms in a concentration-dependent manner.

### ClyQ rescues mice from S. aureus systemic infection.

**FIG 5:**
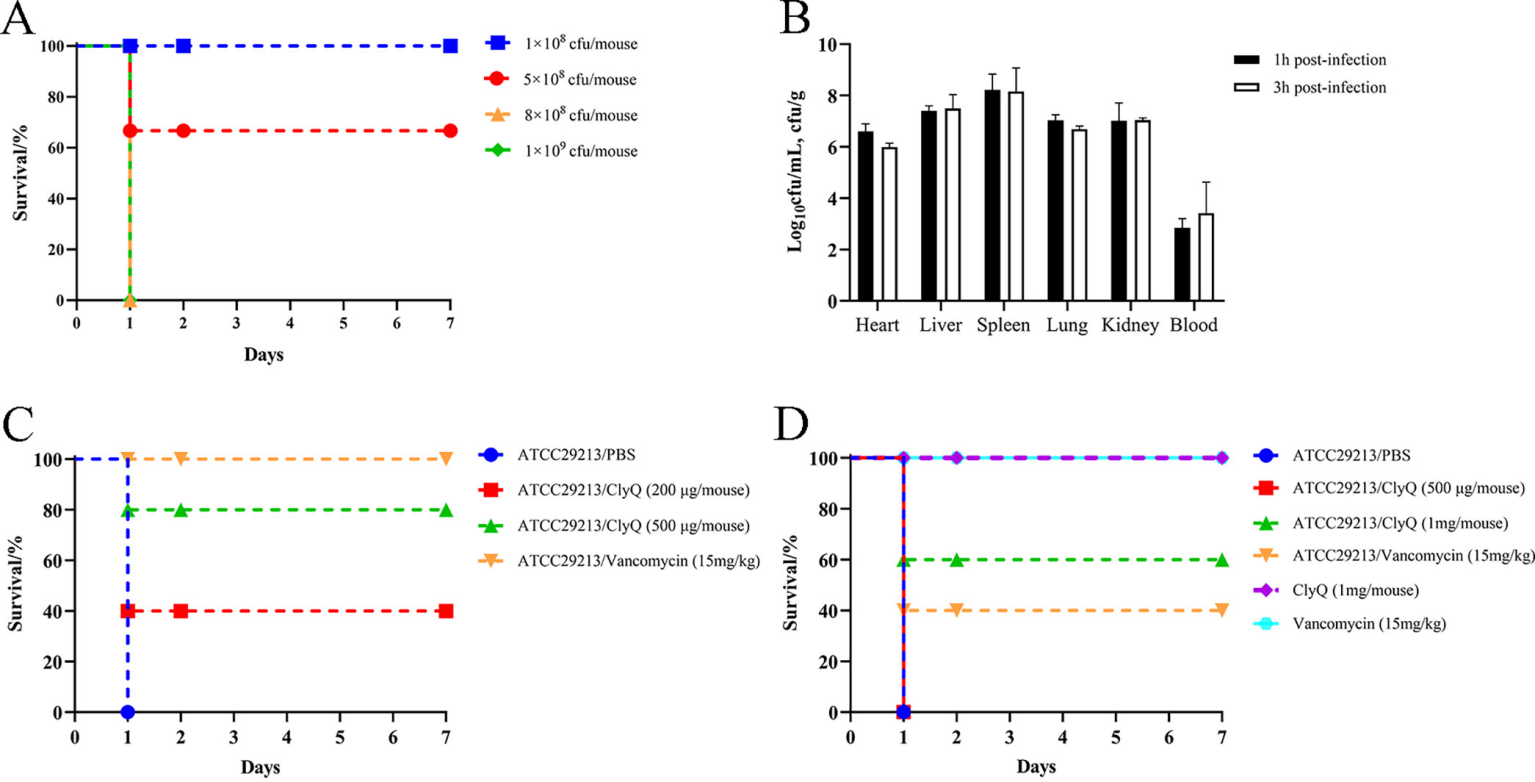
Chimeric lysin ClyQ showed remarkable therapeutic efficiency in the S. aureus systemic infection model. (A) The MLD_100_ of S. aureus ATCC 29213 in mice. The survival of mice infected with different concentrations of S. aureus ATCC 29213 was observed for 7 days. (B) Bacteria abundances in the heart, liver, spleen, lung, kidney, and blood of mice intraperitoneally infected with 1 × MLD_100_ S. aureus ATCC 29213 at 1 h and 3 h postinfection. (C and D) Effect of ClyQ and vancomycin on the survival rate of mice infected with 1 × MLD_100_ S. aureus ATCC 29213. Mice were intraperitoneally treated with PBS, vancomycin (15 mg/kg), and different concentrations of ClyQ at 1 h (C) and 3 h (D) postinfection. The survival rates of mice intraperitoneally injected with ClyQ and vancomycin alone were also recorded for 7 days. Data are expressed as means ± SD.

We identified that 8.0 × 10^8^ CFU/mouse was the minimum lethal dose (MLD_100_) for S. aureus ATCC 29213 (Fig. 5A). Subsequently, a mouse systemic infection model was established by intraperitoneal injection of 1 × MLD_100_ ATCC 29213. At 1 h and 3 h postinfection, a vast amount of S. aureus ATCC 29213 was isolated from the heart (6.60 and 5.99 log), liver (7.41 and 7.50 log), spleen (8.23 and 8.15 log), lung (7.04 and 6.69 log), kidney (7.02 and 7.04 log), and blood (2.84 and 3.42 log) (Fig. 5B). Meanwhile, at 1 h postinfection, the survival rates of mice injected intraperitoneally with 200 μg/mouse ClyQ, 500 μg/mouse ClyQ, and 15 mg/kg vancomycin were 40%, 80%, and 100%, respectively (Fig. 5C). The survival rates of mice treated with 500 μg/mouse ClyQ, 1 mg/mouse ClyQ, and 15 mg/kg vancomycin at 3 h postinfection were 0%, 60%, and 40%, respectively (Fig. 5D).

### A combination of ClyQ and mupirocin exhibits a synergistic bactericidal effect on treating MRSA-induced skin infection.

**FIG 6:**
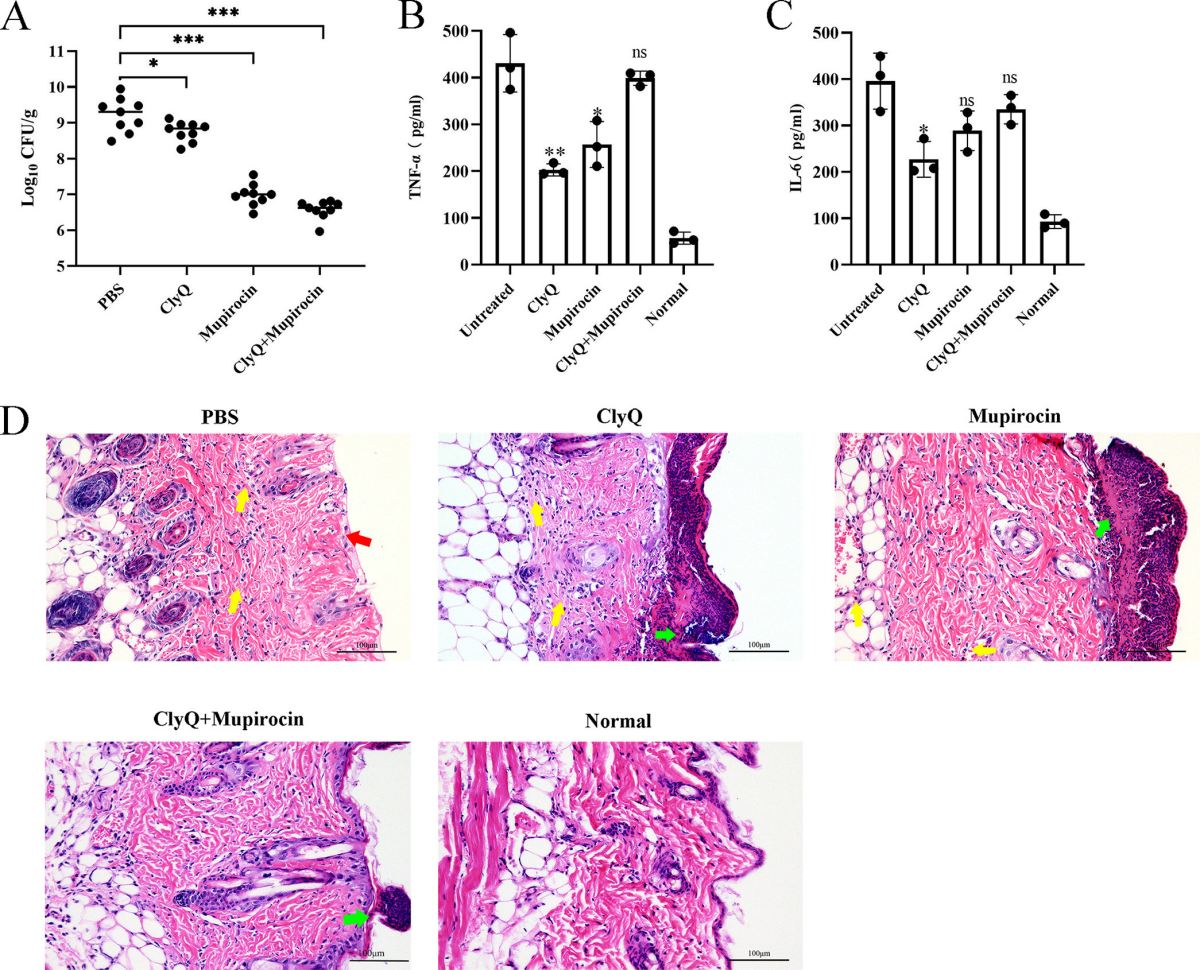
The combination of ClyQ and mupirocin could effectively treat S. aureus-induced skin infection. The four groups of mice were treated with ClyQ, mupirocin, and a combination of ClyQ and mupirocin, respectively, at 20, 23, 26, 36, 39, and 42 h after infection. (A) The bacterial burden of skin tissue was determined at 43 h postinfection. (B and C) Levels of inflammatory cytokines TNF-α (B) and IL-6 (C) in the skin of mice were measured. (D) Hematoxylin and eosin staining of skin samples was observed by microscopic examination (magnification, ×200).

S. aureus, especially MRSA, is a crucial pathogen causing skin infections (22, 23). The S. aureus-induced skin infection model was used in this study to evaluate the bactericidal efficacy of the combination of ClyQ and mupirocin. The results showed that ClyQ, mupirocin, and the combination of ClyQ and mupirocin could decrease the bacteria numbers on skin by 0.46 (*P* < 0.05), 2.23 (*P* < 0.001), and 2.64 (*P* < 0.001) log, respectively, compared to the PBS-treated group (Fig. 6A). A previous study demonstrated that S. aureus skin infection contributes to skin inflammation (24). Here, in the PBS-treated group, the concentrations of tumor necrosis factor-α (TNF-α) and interleukin-6 (IL-6) at the site of S. aureus infection reached 430.69 and 395.79 pg/mL, respectively (Fig. 6B and C). For the control group, the concentrations of TNF-α and IL-6 were 56.8 and 92.9 pg/mL, respectively. Concentrations of TNF-α and IL-6 were significantly decreased by ClyQ treatment compared to in the PBS-treated group. Likewise, mupirocin and a combination of ClyQ and mupirocin could also reduce the levels of TNF-α and IL-6 (Fig. 6B and C).

A histopathological assay was used to evaluate the degree of tissue structure damage. The skin structure of the PBS-treated group was severely abnormal, the dermis was exposed, and the tissue was infiltrated with a high number of inflammatory cells (Fig. 6D). The tissue damage of mupirocin-treated and ClyQ-treated groups was alleviated compared to in the PBS-treated group (Fig. 6D). The combination of ClyQ and mupirocin had the best effect on skin infection. In detail, an abscess in the epidermis was observed, and inflammatory cell infiltration was not significant (Fig. 6D).

**FIG 7:**
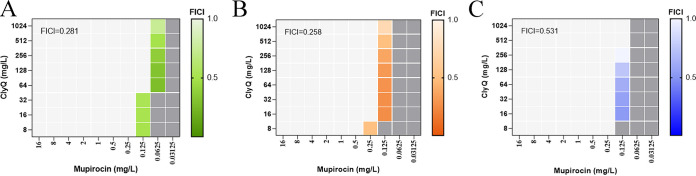
Results of the checkerboard assay for the combination of ClyQ and mupirocin against S. aureus. (A to C) The synergistic effect of the combination of ClyQ and mupirocin against S. aureus S3 (A), ATCC 43300 (B), and ATCC 29213 (C) was tested. PBS was used as a negative control. The FICI values were calculated and are shown in the figures.

Our results showed that the combination of ClyQ and mupirocin had a synergistic antibacterial effect on treating skin infections (Fig. 6A). To determine whether ClyQ and mupirocin have a synergistic bactericidal effect, the fractional inhibitory concentration index (FICI) values of the combination of ClyQ and mupirocin were monitored. The combination of ClyQ and mupirocin against S. aureus S3, ATCC 43300, and ATCC 29213 yielded FICI values of 0.281 (Fig. 7A), 0.258 (Fig. 7B), and 0.531 (Fig. 7C), respectively.

### ClyQ combined with mupirocin delays the development of resistance to mupirocin.

**FIG 8:**
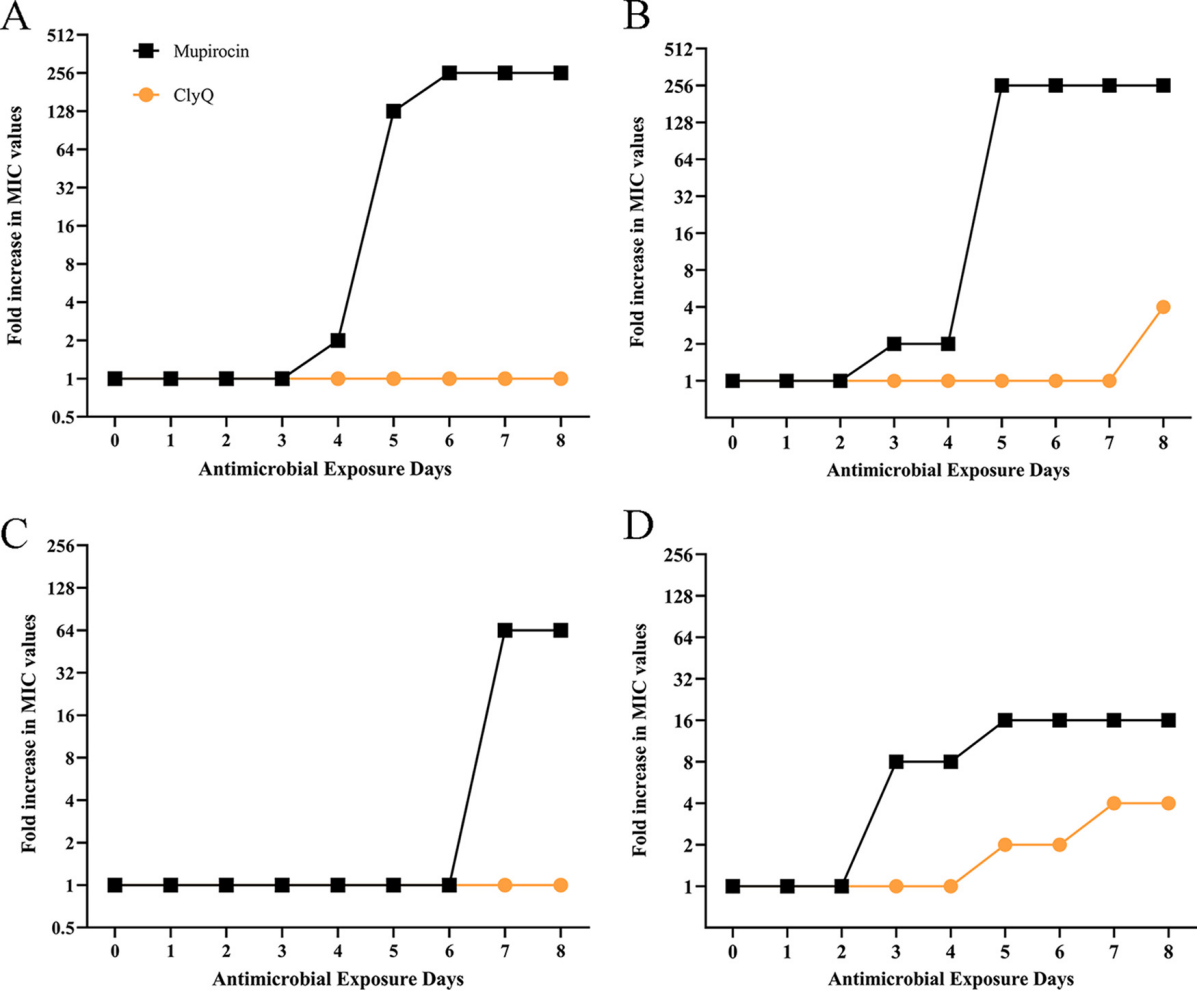
Combination of ClyQ and mupirocin could delay the development of S. aureus resistance. (A and B) MIC changes of ClyQ and mupirocin against S. aureus ATCC 29213 (A) and S3 (B) when S. aureus was exposed to ClyQ or mupirocin alone. (C and D) MIC changes of ClyQ and mupirocin against S. aureus ATCC 29213 (C) and S3 (D) when S. aureus was exposed to the combination of ClyQ and mupirocin.

In the study, we explored the development of resistance of S. aureus ATCC 29213 and S3 to ClyQ and mupirocin. After exposure of the 8th generation of ATCC 29213 to ClyQ, the MIC of ClyQ had not changed (Fig. 8A). After exposure of the 8th generation of S3 to ClyQ, the MIC of ClyQ increased 2-fold (Fig. 8B). In contrast, in the 4th, 5th, and 6th generations of ATCC 29213, the MIC of mupirocin increased surprisingly 2-, 128-, and 256-fold, respectively (Fig. 8A), and in the 3rd and 5th generations of S3, the MIC of mupirocin increased surprisingly 2- and 256-fold, respectively (Fig. 8B). When ClyQ was combined with mupirocin to induce the development of ATCC 29213 resistance *in vitro*, the MIC of ClyQ against ATCC 29213 had not changed in the 8th generation, and the MIC of mupirocin against ATCC 29213 only increased by 64-fold in the 7th generation of ATCC 29213 (Fig. 8C). Similarly, the MIC values of ClyQ in the 5th, 7th, and 8th generations of S3 increased by 2-, 4-, and 4-fold, respectively, and those for mupirocin in the 3rd and 5th generations of S3 only increased by 8- and 16-fold, respectively (Fig. 8D).

## DISCUSSION

In this study, we designed a novel chimeric lysin ClyQ that possesses a broad host range (including staphylococci, streptococci, E. faecalis, and *E. rhusiopathiae*) and excellent antibacterial activity against staphylococci *in vitro*. Similarly, chimeric lysin ClyR can lyse E. faecalis and some species of staphylococci and streptococci (25). However, chimeric lysin ClyS exhibits exceptional muralytic activity against staphylococci (especially S. aureus) but little activity against streptococci (26). Chimeric lysin ClyV possesses lytic activity toward streptococci but not S. aureus and E. faecalis (27). In addition, ClyS is composed of a catalytic domain of S. aureus Twort phage lysin and a CBD from S. aureus phage lysin phiNM3 (26), and ClyR was constructed by fusing the catalytic domain of Streptococcus phage PlyC with a CBD from Streptococcus phage PlySs2 (25). Meanwhile, ClyV was constructed by fusing a catalytic domain of Streptococcus phage lysin PlyGBS with a CBD from E. faecalis phage lysin PlyV12 (27). In contrast, ClyQ was composed of a catalytic domain from S. aureus phage lysin LysGH15 and a CBD of E. faecalis phage lysin PlyV12.

Synergy between phage or phage lysins and antibiotics *in vitro* and *in vivo* has been reported (28). In our study, the combination of chimeric lysin ClyQ and mupirocin demonstrated superiority to ClyQ or mupirocin alone in removing S. aureus from the skin at 43 h postinfection (Fig. 6A). This agrees with other studies that showed that a combination of endolysin MR-10 (50 μg) and minocycline (50 mg/kg orally) is superior to single MR-10 (50 μg) or minocycline (50 mg/kg orally) treatment of MRSA-induced wound infection at 3 h postinfection (10). Combination therapy with Streptococcus phage lysin CF-301 and antibiotics (including daptomycin, vancomycin, and oxacillin) is superior to CF-301 and antibiotic monotherapy for treating S. aureus-induced mouse bacteremia (14). Furthermore, the combination of daptomycin (0.4 mg/kg) and pneumococcal phage lysin Cpl-1 (0.4 mg/kg) significantly increased the percentage of surviving mice infected with S. pneumoniae D39 compared with single daptomycin (0.4 mg/kg) or Cpl-1 (0.4 mg/kg) treatment (29). In contrast, the chimeric lysin ClyS (10% [wt/wt] in Aquaphor) is more effective than mupirocin (2% [wt/wt] in Aquaphor) in removing S. aureus colonized on the skin (12). However, ClyQ also exhibited potent synergy *in vitro*. In contrast to ClyQ and mupirocin monotherapy, the combination of ClyQ and mupirocin could delay the development of resistance to mupirocin (Fig. 8). Previous research has also reported that vancomycin and daptomycin resistance is suppressed by growth in combination with lysin CF-301 (14). To our knowledge, ClyQ is the first chimeric lysin to exhibit the ability to delay antibiotic resistance.

FICI values can determine whether there is a synergistic or adjuvant effect between antibacterial agents (30). Therefore, we measured the FICI values of the combination of chimeric lysin ClyQ and mupirocin. Our results showed that the FICI values of the combination of ClyQ and mupirocin against S. aureus ATCC 43300 and S3 were less than 0.5. However, the FICI values of the combination of chimeric lysin ClyQ and mupirocin against S. aureus ATCC 29213 was 0.531. Similarly, although the superiority of antistaphylococcal endolysin SAL-200 combinations with antibiotics was confirmed when treating S. aureus infection, the effect of the combination of SAL-200 and nafcillin on ATCC 29213 yielded an adjuvant effect (FICI of 0.533) by FICI test (6). In summary, the results indicate that a combination of ClyQ and mupirocin exhibits synergistic antibacterial activity against S. aureus skin infections.

Surprisingly, for ClyQ, mupirocin, and a combination of ClyQ and mupirocin groups, the concentration of cytokines (TNF-α and IL-6) was greatly reduced, while the tendency of decreasing cytokine values was the opposite of the tendency of decreasing visible bacteria numbers (Fig. 6). The cytokine TNF-α, as a proinflammatory cytokine, is produced soon after infection and promotes the acute inflammatory reaction. In the early stages of infection, TNF-α and IL-6 can activate neutrophils (31), and the more rapid lytic activity of lysins may lead to more peptidoglycan fragments, which provoke severe proinflammatory reactions (32). In our previous work, we have also discussed that Toll-like receptors (TLRs) were sensitive to S. aureus lipoproteins and nucleic acids, which could affect the production of proinflammatory cytokines (33). Based on these results, a possible reason could be that the combination of ClyQ and mupirocin could lyse more S. aureus cells than treatment with ClyQ and mupirocin alone, which causes more peptidoglycan fragments, lipoproteins, and nucleic acids to be released. Finally, these peptidoglycan fragments, lipoproteins, and nucleic acids could be recognized by TLRs and produce more TNF-α and IL-6.

Overall, a novel chimeric lysin ClyQ was constructed and showed exceptional antistaphylococcal activity *in vitro* and *in vivo*. ClyQ also possesses the potential as an antimicrobial agent against biofilms formed by S. aureus. Importantly, the combination of ClyQ and mupirocin exhibits synergistic effects in a model of S. aureus-induced skin infections. The combination could delay the development of S. aureus resistance. In conclusion, our findings may enhance the development of new chimeric lysins as antibacterial agents that cooperate with antibiotics to combat S. aureus-induced skin infections and delay resistance development during treatment.

## MATERIALS AND METHODS

### Bacteria strains and culture conditions.

All bacteria strains in this study are listed in Table S1 in the supplemental material. Staphylococcus strains were grown and shaken in lysogeny broth (LB), and the other strains were grown in tryptic soy broth (TSB) containing 5% (vol/vol) bovine serum. Escherichia coli BL21(DE3) was used for protein expression and was grown in LB with kanamycin (50 μg/mL).

### Construction of the chimeric lysin ClyQ.

Genes of the full-length LysGH15 (GenBank: ADG26756.1) and PlyV12 lysins (GenBank: AAT01859.1) were synthesized by Tsingke Biotechnology, Co., Ltd. ClyQ was constructed by fusing the putative CHAP (from LysGH15; 1 to 165 amino acids) with CBD (from PlyV12; 244 to 309 amino acids). The ClyQ gene was digested with BamHI and HindIII and was subsequently ligated into the pET-28a(+) expression plasmid to construct pET-28a(+)-ClyQ. All primers are listed in Table S2.

### Expression and purification of ClyQ.

The plasmid pET-28a(+)-ClyQ was transferred into E. coli BL21(DE3) cells and cultured in LB with 50 μg/mL kanamycin (37°C, 200 × *g*) to logarithmic phase (OD_600_ of ~0.6). The recombinant plasmid was induced by the addition of 0.8 mM isopropyl-β-d-thiogalactopyranoside (IPTG) for 16 to 18 h at 16°C. The induced bacterial solution was centrifuged at 4°C for 10 min (6,000 × *g*) and resuspended with binding buffer (250 mM NaCl, 20 mM Tris-HCl, pH 7.4). The binding buffer containing BL21 cells was broken at high pressure, the broken product was centrifuged at 4°C and 9,000 × *g* for 20 min to remove the broken precipitate, and the supernatant was filtered with a 0.22-μm filter (Biosharp, China). ClyQ was purified by binding the 6×His-tagged fusion protein supernatant to a His-Trap fast flow (FF) column (GE Healthcare Bio-Sciences AB, Uppsala, Sweden) and eluting with elution buffer (250 mM NaCl, 20 mM Tris-HCl, 200 mM imidazole, pH 7.4) according to the manufacturer’s instructions. The purity and molecular weight of the protein were determined by sodium dodecyl sulfate-polyacrylamide gel electrophoresis (SDS-PAGE).

### Characterization of ClyQ.

The thermal stability of ClyQ was determined by incubating ClyQ at 37°C, 40°C, 42.5°C, and 45°C for 1 h. To determine the acid-base stability of ClyQ, different pH buffers (5.0, 6.0, 7.0, 8.0, 9.0, 10.0, and 11.0) were mixed with ClyQ at 37°C for 1 h. When incubating at different temperatures or pH environments, 100 μL of ClyQ (100 μg/mL) was taken out every 10 min and added into the same volume of S. aureus S3. After incubation at 37°C for 30 min, the OD_600_ of the mixture was monitored. Meanwhile, the thermal stability of ClyQ (500 μg/mL) was monitored by nano-differential scanning fluorimetry (nanoDSF) using a Prometheus NT.48 instrument from NanoTemper Technologies (34). The metal ion stability of ClyQ was determined by incubating in PBS buffer (137 mM NaCl, 2.7 mM KCl, 4.3 mM Na_2_HPO_4_·H_2_O, and 1.4 mM KH_2_PO_4_, pH 7.4) supplemented with 10, 100, and 1,000 μM Ca^2+^, Mg^2+^, Zn^2+^, Ba^2+^, and Mn^2+^ for 1 h at 37°C; 100 μL of ClyQ (100 μg/mL) was then mixed with 100 μL of bacteria in 96-well plates. The plates were incubated at 37°C for 30 min, and the OD_600_ was recorded.

### Bactericidal assays.

The host range of ClyQ and LysGH15 was determined by the turbidity reduction method (13, 35). Briefly, 100 μL of bacterial suspension and 100 μL of ClyQ or LysGH15 with a concentration of 100 μg/mL were mixed in a 96-well plate, and the OD_600_ values of the mixtures were measured after incubation at 37°C for 30 min. For the dose-dependent assay, S. aureus ATCC 43300 and S3 were mixed with ClyQ at final concentrations ranging from 0.78 μg/mL to 50 μg/mL, and the mixture was incubated at 37°C for 1 h. The OD_600_ was measured every 5 min, and PBS buffer was used to suspend bacteria and ClyQ.

To evaluate the antistaphylococcal activity of ClyQ and LysGH15, the number of bacterial colonies was calculated by mixing 100 μL of bacterial suspension with 100 μL of ClyQ and LysGH15 (100 μg/mL), and the mixture was incubated at 37°C for 1 h. Moreover, ClyQ was mixed with S. aureus ATCC 43300 and S3 and incubated at 37°C for 60 min, and bacterial cells were monitored at 20-min intervals. Bacterial cell numbers were calculated by plate counting of a 10-fold serial dilution.

### MIC of ClyQ.

The broth dilution method was used to determine the MIC of ClyQ (36). Bacteria were diluted in 0.9% normal saline to a standard of 0.5 McIntosh turbidity and further diluted to 1 × 10^5^ CFU/mL by the addition of cation-adjusted Mueller-Hinton broth (CAMHB; Becton, Dickinson, Franklin Lakes, NJ) (37). Then, 100 μL of bacteria suspension was added with 100 μL of ClyQ at different concentrations (1 to 512 mg/L) in 96-well plates. The results were observed after incubation at 37°C for 18 h. The MIC was defined as the lowest protein concentration inhibiting visible bacteria growth. The experiment was repeated three times.

### Antibiofilm activity of ClyQ.

The 96-well plates were used to test the antibiofilm ability of ClyQ, as previously described (33, 38). The bacteria concentration was adjusted to 1 × 10^6^ CFU/mL, and 200 μL of the bacteria solution was added to 96-well plates and incubated for 24 or 48 h at 30°C. Subsequently, the biofilms were treated with 200 μL of ClyQ (0, 5, and 40 μg/mL) at 37°C for 1 h. Finally, the biomass of biofilms was determined by crystal violet (1.0% [wt/vol]) staining, and bacterial burden in biofilms was determined by plate counting of a 10-fold serial dilution.

### SEM images of S. aureus biofilms.

The biofilms of S. aureus S3 and ATCC 43300 were grown in 500 μL of LB medium on 24-well plates with coverslips at 30°C for 48 h. The 24-well plates were then treated with ClyQ at a concentration of 40 μg/mL at 37°C for 1 h, and PBS was used as a negative control. After the treatment, plates were washed twice with PBS, fixed with 2% glutaraldehyde for 2 h, and washed twice with PBS. Finally, the coverslips were sprayed with gold, and images of S. aureus were acquired by FESEM (magnifications of ×2,000 and ×5,000; NTC, Japan).

### Ethics statement.

All animal experiments were approved by the Laboratory Animal Monitoring Committee of Huazhong Agricultural University and were performed according to the corresponding guidelines for laboratory animal operations in Huazhong Agricultural University. The corresponding ethical approval code is HZAUMO-2022-0057.

### Mouse infection model.

The systemic infection models were performed as described previously (16, 39). Specific pathogen-free (SPF) BALB/c female mice (6 weeks old) were purchased from the Experimental Animal Centre of Huazhong Agricultural University, China. First, different concentrations of S. aureus ATCC 29213 (1 × 10^8^, 5 × 10^8^, 8 × 10^8^, and 1 × 10^9^ CFU/mouse) were injected intraperitoneally to determine the MLD_100_ of ATCC 29213. In the mouse systemic infection model, ATCC 29213 was injected intraperitoneally (8 × 10^8^ CFU/mouse; 1 × MLD_100_), and organs (heart, liver, spleen, lung, and kidney) were weighed and homogenized in 1 mL of PBS for 5 min at 60 reps (Retsch MM400) at 1 h and 3 h after the challenge. Blood and homogenates were serially diluted and plated on LB agar for the assessment of bacterial abundance. Plates were incubated at 37°C overnight, and numbers of visible bacteria were counted. For survival rate experiments, each group (*n* = 5) of mice was intraperitoneally injected with different concentrations of ClyQ (200 and 500 μg/mouse), 15 mg/kg vancomycin, or PBS (200 μL) at 1 h postinfection, and mice were intraperitoneally injected with various concentrations of ClyQ (500 μg/mouse and 1 mg/mouse), 15 mg/kg vancomycin, or PBS (200 μL) at 3 h postinfection. Uninfected mice were intraperitoneally injected with 1 mg/mouse ClyQ (*n* = 5) and 15 mg/kg vancomycin (*n* = 5) as the negative control. Mouse survival rates were observed for 7 days.

### Mouse skin infection model.

A mouse model of skin infection was performed as described previously, with minor modifications (40). An area of skin (approximately 2 cm^2^) on the dorsal side of each mouse was stripped using 3M autoclaved tapes 20 times. Mouse skin was treated with PBS as a vehicle control and with 1 μg/mouse mupirocin (2%, 50 μL) as a positive control (12). The concentration of S. aureus S3 was controlled to 5 × 10^8^ CFU/mL, and 20 μL (1 × 10^7^ CFU) of the bacterial solution was added to the wound area. Subsequently, the mice were divided into four groups (*n* = 9) and treated with PBS, ClyQ (50 μg/mouse), mupirocin (1 μg/mouse), and the combination of ClyQ (50 μg/mouse) and mupirocin (1 μg/mouse), respectively, at 20, 23, 26, 36, 39, and 42 h after infection. Mice were euthanized at 43 h postinfection, and the injured skin was homogenized. Homogenates were serially diluted and plated on LB agar plates for visible cell counting. Simultaneously, the skin samples were fixed with a 4% paraformaldehyde solution for histopathological assessment. Concentrations of TNF-α and IL-6 in the skin tissue were determined using Quantikine mouse TNF-α/IL-6 commercial kits (NeoBioscience, Inc., Shenzhen, China).

### Checkerboard assay.

The checkerboard assay for the combination of ClyQ and mupirocin was performed in 96-well plates (6). Concentration gradients between ClyQ and mupirocin were prepared in the horizontal and vertical directions, and PBS was used as a negative control. The method of preparing bacteria was the same as for MIC determination, as described above, and the bacteria were added to each well. The plates were incubated at 37°C for 18 h. Subsequently, the MIC was recorded. The values of the fractional inhibitory concentration index (FICI) were calculated according to the previous literature (30).

### Resistance development assay.

The development of bacterial resistance to ClyQ and mupirocin was tested according to previous methods, with minor modifications (12, 41, 42). S. aureus S3 and ATCC 29213 were exposed to increasing concentrations (1/32 × MIC to 4 × MIC) of ClyQ, mupirocin, and a combination of ClyQ and mupirocin. Each culture generation was divided into two aliquots. One aliquot received CAMHB with the next concentration of ClyQ, mupirocin, and a combination of ClyQ and mupirocin and was incubated at 37°C for 12 h. The other aliquot was plated on MHB agar plates that were treated with 1 × MIC of ClyQ, mupirocin, and a combination of ClyQ and mupirocin. Three colonies grown on plates were used to determine the MIC values of ClyQ, mupirocin, and a combination of ClyQ and mupirocin. The process was repeated for 8 rounds.

### Statistical analysis.

The experimental data are presented as mean ± standard deviation (SD). Statistical significance was determined using an unpaired Student’s *t* test. A *P* value of <0.05 was considered a statistically significant difference. GraphPad Prism (version 9) was used for all statistical analyses.

## Supplementary Material

Reviewer comments
