# Supplementary material for: Exploiting Broad-Spectrum Chimeric Lysin to Cooperate with Mupirocin against Staphylococcus aureus-Induced Skin Infections and Delay the Development of Mupirocin Resistance
Source: Microbiol Spectr. 2023 May 1;11(3):e05050-22. doi: 10.1128/spectrum.05050-22 (PMC10269905; doi:10.1128/spectrum.05050-22)
Supplement: Supplemental file 1 — Supplemental material. Download spectrum.05050-22-s0001.pdf, PDF file, 0.4 MB [file spectrum.05050-22-s0001.pdf]

Supplement data

Supplement figures

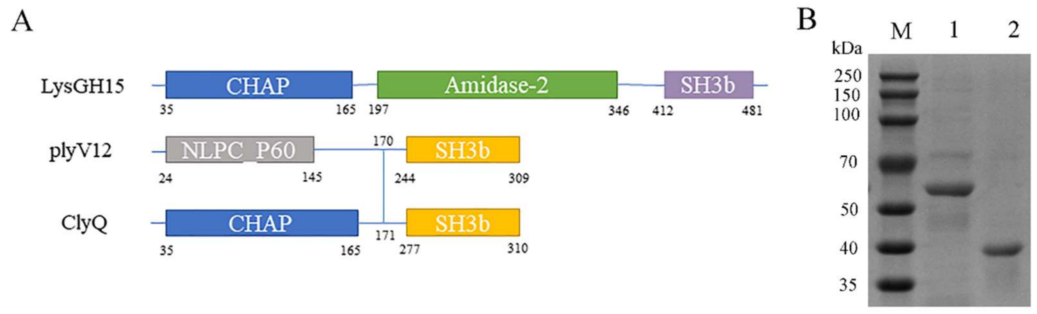

Fig. S1. General features of ClyQ. (A) Schematic representation of Chimeric lysin ClyQ. The ClyQ was constructed by fusing the putative CHAP (from LysGH15, 1-165aa) and CBD (from PlyV12, 244-309aa). (B) SDS-PAGE analysis of the recombinant protein ClyQ (M: marker; 1: purified LysGH15; 2: purified ClyQ).

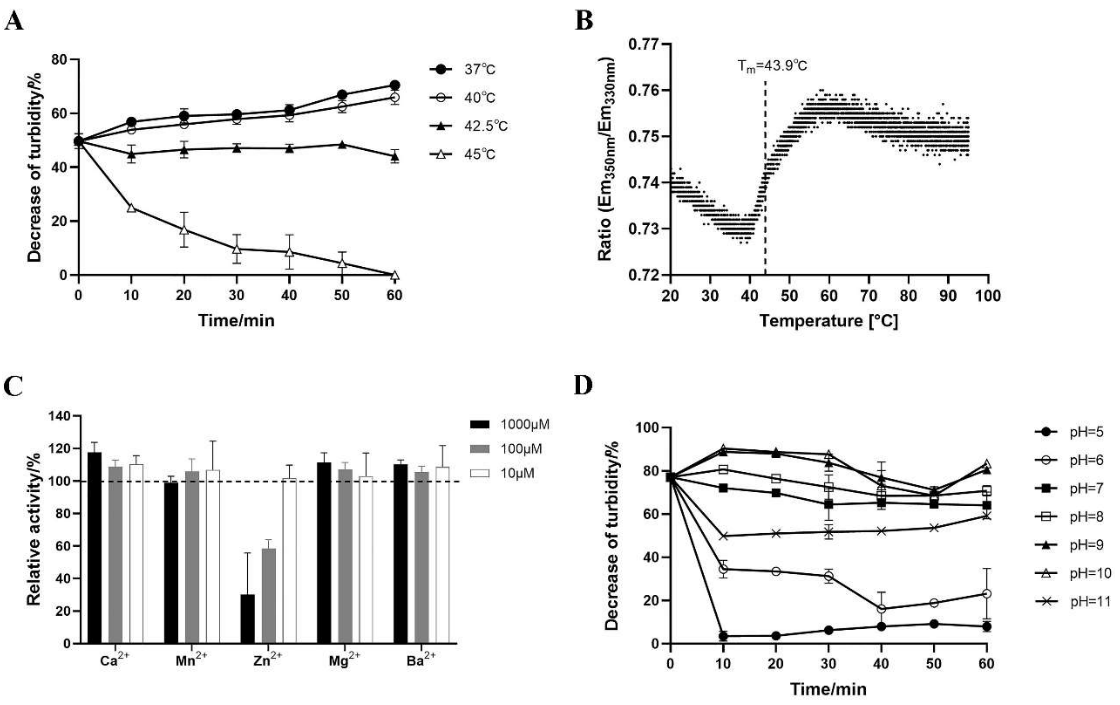

Fig. S2. Characterization of ClyQ. (A) The lytic activity of ClyQ was affected by different temperatures from 37 to 45°C. (B) The intensity of TRP emission at 330 and

350 nm was recorded to monitor changes in protein 3D structure by NanoDSF. (C) The effects of Ca^{2+}, Mg^{2+}, Zn^{2+}, Ba^{2+}, and Mn^{2+} on the activity of ClyQ. (D) The activity of ClyQ in different pH environments. Error bars are expressed as means ± the standard deviations (SD).

Supplement tables

Table S1. The strains used in this study

Species	Strains	Origin
S. aureus (MRSA)	S3	Human
S. aureus (MRSA)	S6	Human
S. aureus (MSSA)	S8	Human
S. aureus (MSSA)	S13	Swine
S. aureus (MSSA)	S20	Swine
S. aureus (MRSA)	S21	Swine
S. aureus (MRSA)	S22	Swine
S. aureus (MSSA)	S27	Swine
S. aureus	785	Swine
S. aureus	538	Swine
S. sciuri	768	Swine
S. simulans	Z7	Swine
S. simulans	Z27	Swine
S. simulans	852	Swine
S. cohnii	Z10	Swine
S. cohnii	Z18	Swine
S. cohnii	863	Swine
S. epidermidis	Z17	Swine
S. epidermidis	Z21	Swine
S. heamolyticus	Z19	Swine
S. heamolyticus	774	Swine
S. heamolyticus	638	Swine
S. warneri	Z20	Swine
S. chromogenes	Z4-1	Swine
S. chromogenes	Z9-1	Swine
S. chromogenes	H10-3	Swine
S. xylosus	019-1	Swine
S. xylosus	005-2	Swine
S. hyicus	st6-1	Swine

S. hyicus	st85	Swine
S. muscae	st84	Swine
S. nepalensis	st47	Swine
S. equorum	st39	Swine
S. equorum	st40	Swine
S. rostri	st17	Swine
S. saprophyticus	st3	Swine
S. saprophyticus	st32	Swine
S. arlettae	st57	Swine
S. agalactiae	5374	Bovine
S. agalactiae	X2	Bovine
S. dysgalactiae	ATCC13813	Bovine
S. dysgalactiae	T1	Bovine
S. dysgalactiae	H1-1	Bovine
S. uberis	2	Bovine
S. uberis	009-1	Bovine
S. suis	SC19	Bovine
S. suis	859	Bovine
E. faecalis	012-2	Bovine
E. faecalis	004-2	Bovine
E. faecalis	009-2	Bovine
E. rhusiopathiae	1801	Bovine
S. aureus (MRSA)	ATCC43300	
S. aureus (MSSA)	ATCC29213	
L. Monocytogenes	ATCC19111	
S. typhimurium	ATCC14028	
E. Coli	ATCC25922	
E. Coli	BL21 (DE3)	

Table S2. Primers used in the study.

Primers	Sequences (5'-3')
LysGH15CHAP-F	GGATCCATGGCAAAAACACAAGCTGA
LysGH15CHAP-R	CGATCCAGACGAGCCTCCGGCTTTAACCGGGATC
PlyV12CBD-F	GGAGGCTCGTCTGGATCGCTAAATGGGGGATCAACA
PlyV12CBD-R	AAGCTTTTATTTGAAAGTACCCCA

The restriction sites and linker are underlined.
*Bam*HI: GGATCC, *Hin*dIII: AAGCTT, linker: GGAGGCTCGTCTGGATCG.
